# Chilean version of the INECO Frontal Screening (IFS-Ch): psychometric properties and diagnostic accuracy

**DOI:** 10.1590/S1980-57642013DN70100007

**Published:** 2013

**Authors:** Josefina Ihnen Jory, Andrés Antivilo Bruna, Carlos Muñoz-Neira, Andrea Slachevsky Chonchol

**Affiliations:** 1 Unidad de Neurología Cognitiva y Demencias, Servicio de Neurología, Hospital del Salvador. Santiago, Región Metropolitana, Chile. Universidad de Chile.; 2 Departamento de Psicología, Facultad de Ciencias Sociales, Universidad de Chile. Departamento de Psicología, Facultad de Ciencias Sociales, Universidad de Chile.; 3 Unidad de Neurología Cognitiva y Demencias, Servicio de Neurología, Hospital del Salvador. Santiago, Región Metropolitana, Chile. Universidad de Chile. Departamento de Psicología, Facultad de Ciencias Sociales, Universidad de Chile. Centro de Investigación Avanzada en Educación, Universidad de Chile. Departamento de Farmacología Molecular y Clínica, ICBM y Departamento de Ciencias Neurológicas Oriente, Facultad de Medicina, Universidad de Chile, Santiago, Chile. Servicio de Neurología, Clínica Alemana de Santiago, Región Metropolitana, Chile.

**Keywords:** INECO frontal screening, executive functions, neuropsychological tests, dementia

## Abstract

**OBJECTIVE:**

This study sought to analyze the psychometric properties and diagnostic accuracy of the Chilean version of the INECO Frontal Screening (IFS-Ch) in a sample of dementia patients and control subjects.

**METHODS:**

After adapting the instrument to the Chilean context and obtaining content validity evidence through expert consultation, the IFS-Ch was administered to 31 dementia patients and 30 control subjects together with other executive assessments (Frontal Assessment Battery [FAB], Modified version of the Wisconsin Card Sorting Test [MCST], phonemic verbal fluencies [letters A and P] and semantic verbal fluency [animals]) and global cognitive efficiency tests (Mini mental State Examination [MMSE] and Addenbrooke's Cognitive Examination-Revised [ACE-R]). Caregivers of dementia patients and proxies of control subjects were interviewed with instruments measuring dysexecutive symptoms (Dysexecutive Questionnaire [DEX]), dementia severity (Clinical Dementia Rating Scale [CDR]) and functional status in activities of daily living (Activities of Daily Living Scale [IADL] and Technology-Activities of Daily Living Questionnaire [T-ADLQ]). Convergent and discriminant validity, internal consistency reliability, cut-off points, sensitivity and specificity for the IFS-Ch were estimated.

**RESULTS:**

Evidence of content validity was obtained. Evidence of convergent validity was also found showing significant correlations (p<0.05) between the IFS-Ch and the other instruments measuring: executive functions (FAB, r=0.935; categories achieved in the MCST, r=0.791; perseverative errors in the MCST, r= -0.617; animal verbal fluency, r=0.728; "A" verbal fluency, r=0.681; and "P" verbal fluency, r=0.783), dysexecutive symptoms in daily living (DEX, r= -0.494), dementia severity (CDR, r= -0.75) and functional status in activities of daily living (T-ADLQ, r= -0.745; IADL, r=0.717). Regarding reliability, a Cronbach's alpha coefficient of 0.905 was obtained. For diagnostic accuracy, a cut-off point of 18 points (sensitivity=0.903; specificity=0.867) and an area under curve of 0.951 were estimated to distinguish between patients with dementia and control subjects.

**DISCUSSION:**

The IFS-Ch showed acceptable psychometric properties, supported by evidence of validity and reliability for its use in the measurement of executive functions in patients with dementia. The diagnostic accuracy of the IFS-Ch for detecting dementia patients was also considered acceptable.

## Abstract (pt)

**OBJETIVO:**

Analisar as propriedades psicométricas e utilitário de diagnóstico da versão chilena do rastreio frontal INECO (IFS-Ch) em uma amostra de pacientes com demência e controles.

**MÉTODOS:**

Após a adaptação do instrumento para o contexto chileno e obtenção de evidências de validade de conteúdo, o IFS-Ch foi administrado a 31 pacientes com demência e 30 indivíduos do grupo controle, além de outros testes de eficiência cognitiva global e executiva. Cuidadores de pacientes com demência e informantes de indivíduos controles foram entrevistados com instrumentos de medidas de sintomas disexecutivos, gravidade da demência e estado funcional nas atividades da vida diária. Validade convergente e discriminante, consistência interna, pontos de corte, sensibilidade e especificidade para o IFS-Ch foram estimados.

**RESULTADOS:**

A evidência de validade de conteúdo foi obtida através de consulta a um especialista. Evidências de validade convergente foram encontrados, bem como, descritas correlações significativas entre o IFS-Ch e outros instrumentos de medidas: de funções executivas (FAB, r=0,935; categorias alcançadas no MCST, r=0,791; erros perseverativos na MCST, r= -0,617; fluência verbal animais, r=0,728; "A" de fluência verbal, r=0,681; gravidade de demência e fluência verbal de "P", r=0,783), sintomas disexecutivos na vida diária (DEX, r= -0,494), (CDR, r= -0,75) e estado funcional nas atividades da vida diária (T-ADLQ, r= -0,745; AIVD, r=0,717). Quanto à confiabilidade, coeficiente alfa de Cronbach de 0,905 foi obtido. Quanto a utilidade de diagnóstico, um ponto de corte de 18 pontos (sensibilidade=0,903, especificidade=0,867) e uma área sob a curva de 0,951 foi estimada para distinguir entre pacientes com demência e sujeitos controles.

**DISCUSSÃO:**

O IFS-Ch mostra propriedades psicométricas aceitáveis, apoiadas por evidências de validade e confiabilidade para sua utilização como medida de funções executivas em pacientes com demência. Sua utilidade diagnóstica para detectar pacientes com demência também é considerada aceitável.

## INTRODUCTION

Executive functions constitute a group of higher order abilities that coordinate basic cognitive processes in order to regulate, control and execute goal-oriented behaviors that require new and creative solutions.^1-3^ These include a wide range of cognitive processes such as inhibition, working memory, shifting, verbal reasoning, multitasking and planning,^4,5^ all of which involve significant activity of the frontal lobes and "frontal lobe systems", i. e. those areas with direct connections with the frontal lobes.^6^

This cognitive domain is impaired in numerous neurological and neuropsychiatric pathologies, such as focal lesions involving the frontal lobes (abscesses, strokes or tumors), inflammatory diseases, neurodegenerative disorders, schizophrenia, obsessive compulsive disorder, etc.^7^ Executive dysfunction has also been observed early in most types of dementia, to the point where some authors have defined it as its core symptom.^8^ Accordingly, the assessment of executive functions contributes to an early diagnosis of dementia. Moreover, executive deficits are prominent symptoms of some dementia syndromes, such as frontotemporal dementia (behavioral variant)^9^ and vascular dementia^10,11^ Hence, the assessment of this cognitive domain also contributes to the differential diagnosis of the specific type of dementia.

**Figure 1:**
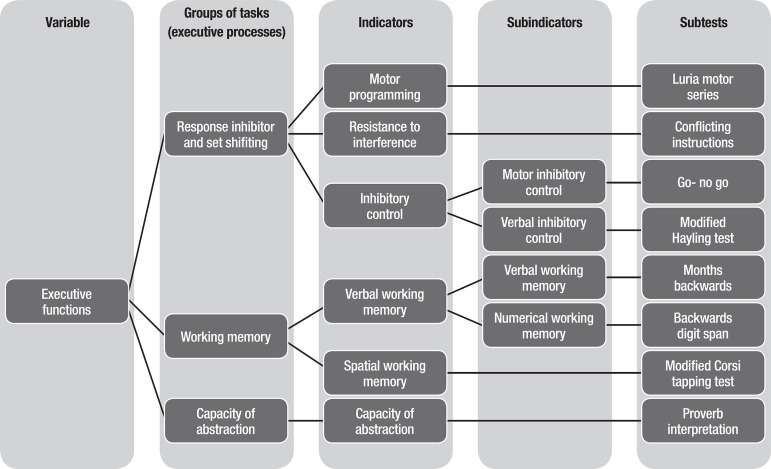
Structure of the INECO Frontal Screening.

The above-mentioned facts, together with the high and increasing prevalence of dementia,^12^ have prompted the development of executive screening tests to be applied in neurological and general medical practice with elderly patients that can provide brief and quick assessment of this cognitive domain. The INECO Frontal Screening (IFS) is an executive screening test that assesses several executive processes using a few tasks.^13^ It comprises three of the subtests included in the Frontal Assessment Battery (FAB) - another executive screening test that has shown good characteristics for assessing executive dysfunctions:^14,15^ those which have shown the highest sensitivity according to the test author's everyday clinical experience^13^ as well as empirical evidence^16^ (Luria Motor Series, Conflicting Instructions and Go-no go). In addition, the IFS includes new subtests, most of them assessing various dimensions of working memory. Figure 1 shows the detailed structure of the IFS, describes the variables assessed by the test, its indicators and sub-indicators, and the subtests that measure each indicator or subindicator.

Since the IFS has only been validated in Argentina and to the best of our knowledge neither content validity nor correlation of the IFS with functionality and dysexecutive behaviors in daily living have been examined, it would be valuable to consider these aspects in order to complement the study of the instrument. Therefore, the aim of the present study was to adapt the IFS to the Chilean cultural context and evaluate its psychometric properties and diagnostic accuracy in a sample of control subjects and dementia patients.

## METHODS

**Subjects.** The study was carried out in a convenience sample, which included participants of both sexes, Spanish speakers, aged 52 or older, with at least three years of formal education. All subjects had a proxy that gave relevant information about their everyday activities and behavior. Subjects were divided into two groups:

A clinical sample, including 31 patients recruited from the Cognitive Neurology and Dementias Unit (Unidad de Neurología Cognitiva y Demencias) of the Neurology Service at the Hospital del Salvador in Santiago, Chile. The diagnosis of dementia was provided by a neurologist based on detailed neurological, neuropsychological, laboratory, and neuroimaging data from each participant. The first step in the diagnostic process was to determine the presence of dementia using the DSM-IV-TR criteria.^17^ When these criteria were met, the Neurologist determined the specific types of dementia using multiple diagnostic criteria for AD (i. e., NINCDS-ADRDA), vascular dementia (i. e., ADDTC, NINDS-AIREN), Dementia with Lewy Bodies (i. e., third report of the DLB Consortium) or frontotemporal dementia (i. e, Consensus for FTD diagnosis).^18-21^ All patients had a Clinical Dementia Rating Scale (CDR) ≥1. More specifically, 10 patients had AD, 3 with VD, 2 with mixed dementia, 5 with LBD, 5 with bvFTD, 2 with SD and 4 dementia patients with non-specified etiology, were included in the sample.

The control sample comprised 30 subjects with similar socio-demographic characteristics (age, gender and years of education) to those of the clinical sample. All participants included in this group had CDR=0 and presented no symptoms or history of neurological or psychiatric diseases.

Finally, exclusion criteria for both groups were: [1] presence of depression as measured by the Geriatric Depression Scale (score ≤5 points); [2] presence of Anxiety Disorder as measured by the Zung Scale (score ≤51 points); and [3] presence of severe sensory deficits (loss of vision and/or hearing) that could impede test administration.

**IFS and other neuropsychological tests.** As outlined above, the IFS is a screening test for executive dysfunctions. The tasks included in the IFS are: Luria motor series (3 points), Conflicting instructions (3 points), Go-no go (3 points), Months backwards (2 points), Backwards digit span (6 points), Modified Corsi tapping test (4 points), Proverb interpretation (3 points) and Modified Hayling Test (6 points). Thus, the IFS has a maximum possible score of 30 points. High scores indicate preservation of the executive functions. In this study, the IFS was adapted to the Chilean cultural context (IFS-Ch) and then administered to all subjects.

All subjects were assessed with the following executive tests to estimate convergent validity. [1] The Modified version of the Wisconsin Card Sorting Test (MCST),^22^ a brief version of the widely known Wisconsin Card Sorting Test^23,24^ designed originally to study "abstract behavior" and "set-shifting ability" and later proposed as being sensitive for assessing frontal damage.^24^ The MCST is a classification task in which the subject must find the sorting criteria and maintain it for a number of trials.^14^ This particular version was used as it simplifies and reduces ambiguity in administration, making it more suitable for elderly patients.^25^ [2] Verbal fluency tasks, or controlled oral word-association, in which subjects have to generate words following a given criteria. This test is sensitive for assessing executive dysfunction^24,26^ and semantic memory impairment.^24^ Semantic verbal fluency (animals) and phonemic verbal fluencies (letters A and P)^27^ were specifically used. [3] The FAB, a screening test for executive dysfunction that assesses conceptualization, mental flexibility, motor programming, resistance to interference, inhibitory control and environmental autonomy.^14^

All participants were also tested with global cognitive efficiency measures: [1] the Mini Mental State Examination (MMSE),^28^ the most commonly used cognitive screening test internationally;^29^ and [2] the Addenbrooke's Cognitive Examination Revised- Chilean Version (ACE-R-Ch),^30^ a test that assesses five cognitive domains: orientation and attention, memory, verbal fluency, language and visuospatial abilities.

Proxies were interviewed with instruments to assess dysexecutive symptoms in daily life (Dysexecutive Questionnaire [DEX]),^31^ dementia severity (CDR)^32^ and functional capacity in activities of daily living (Instrumental Activities of Daily Living Scale (IADL)^33^ and Technology-Activities of Daily Living Questionnaire (T-ADLQ)).^34^

**Procedure.** The IFS was first adapted to the Chilean cultural context and its content validity was assessed by consultation with experts through a content validity questionnaire. All subjects were assessed by the modified IFS (IFS-Ch) and the other instruments previously described.

**Statistical analysis.** All statistical analyses were performed with significance level set at 0.05. Data analysis was performed with PASW Statistics 18 software. Differences in gender were analyzed using the χ^2^; test. Differences in age, years of education and test scores between groups were analyzed using the t test for independent samples. A one-way MANOVA analysis was conducted to compare results across subtests of the IFS-Ch by diagnostic category. The correlations between scores of the two tests were evaluated using the Pearson coefficient, with the exception of the association between CDR and IFS-Ch scores, for which the Spearman rank correlation test was employed. Reliability was assessed using the Cronbach's alpha coefficient. The sensitivity and specificity of the IFS-Ch for detecting the presence of dementia were evaluated using the receiver operating characteristic (ROC) analysis.

**Ethical concerns.** The study was approved by the Ethics Committee at the Servicio de Salud Metropolitano Oriente. Informed consent was obtained from control subjects, dementia patients and their closest relatives.

## RESULTS

**Adaptation.** Given its sociocultural nature, the proverb interpretation subtest of the IFS was adapted to the Chilean cultural context. Using a four-point Likert scale, six experts in the neuropsychological field were consulted about the capacity of the three proverbs included in the original test and three proverbs proposed as relatively common in Chile to assess executive function and their level of familiarity in the Chilean cultural context. The three proverbs that presented the highest means and the lowest standard deviations were selected. Table 1 summarizes the statistical parameters for the experts' responses. Only minor modifications were made to the rest of the test administration procedure and scoring instructions in order to standardize the assessment procedure as much as possible.

**Table 1:** Mean and standard deviation of the proverbs' capacity to assess executive functions and level of familiarity in Chilean population.

Proverbs	Capacity to assess executive functions	Level of familiarity in Chile
Perro que ladra no muerde	3.67±0.516	3.67±0.516
A mal tiempo, buena cara	3.5±0.837	3.83±0.408
En casa de herrero, cuchillo de palo	3.83±0.408	3.67±0.516
Más vale pájaro en la mano que cien volando	3.83±0.408	3.67±0.516
Camarón que se duerme se lo lleva la corriente	3.67±0.516	3.5±0.548
Si el río suena es porque piedras trae	3.67±0.516	3.33±0.516

Results expressed in Mean±Standard Deviation. The selected proverbs are highlighted in bold letters.

**Demographic and neuropsychological data.** Table 2 shows demographic and neuropsychological data for the clinical and control samples. No significant differences in gender, age or years of education were found among the groups (p>0.05). In contrast, the scores of all the instruments administered to subjects and their informants differed significantly between the groups studied (p<0.05).

**Table 2:** Demographic and neuropsychological data.

Parameters		Descriptive statistics by group		Comparison Significance
		**Control (n=30)**	**Dementia (n=31)**	
Age		70.9±8.2	74.1±9.2	n.s.
Years of education		11.9±4.5	9.7±4.7	n.s.
Sex*	%Men (n)	46.7% (14)	54.8% (17)	n.s.
	%Women(n)	53.3% (16)	45.2% (14)	
IFS-Ch		21.7±3.4	9.8±5.7	**
FAB		16±1.6	9.1±3.9	**
MCST (categories achieved)		4.9±1.4	2.2±1.4	**
MCST (perseverative errors)		2.5±3.1	7.7±4.5	**
"A" verbal fluency		11.7±4.4	5.3±4.3	**
"P" verbal fluency		15.1±4.5	6.6±5.2	**
Animals verbal fluency		17.2±5.4	4.8±3.1	**
DEX		8.7±8.3	33.8±16.2	**
CDR		0±0	1.6±0.8	**
IADL		7.7±0.6	3.5±1.9	**
T-ADLQ		4.1±5.9	49.5±18.8	**
ACE-R-Ch		90.9±7.1	49.4±18.7	**
MMSE		28.9±1.3	18.1±6.6	**

Results expressed in Mean±Standard Deviation.

* Chi-Square, all other comparisons were carried out with a t test for independent samples.

** Significant difference, p<0.05.

n.s. non significant difference, p>0.05.

**Influence of socio-demographic variables on IFS-Ch performance.** In order to determine the influence of demographic variables on IFS-Ch performance, the correlation between demographic characteristics and IFS-Ch total scores was estimated. No significant association was found between IFS-Ch total scores and age (r= -0.197; p>0.05), whereas a significant correlation was found between IFS total scores and years of education (r=0.48; p<0.001). Regarding sex, no significant gender differences were found on IFS performance (t= -0.25; p>0.05). In summary, only years of education showed an influence on IFS performance.

**Evidence of validity.** *Content validity* - Five experts with at least two years of experience in the field of neuropsychology answered a content validity questionnaire designed for the IFS-Ch. In this questionnaire, the conceptual and operational definitions of executive functions and its indicators were presented. The definition of each indicator was followed by the administration and scoring instructions for the corresponding subtest. Subsequently, the experts were asked about the capacity of each subtest to assess executive function, its capacity to measure the corresponding indicator, and the clarity of the administration and scoring instructions, leaving a space for any other observations. All the experts agreed that each of the subtests measured executive functions and that each subtest assessed its respective indicator. For 5 of the 8 subtests, all the experts considered that the instructions were formulated clearly, while for the 3 remaining subtests, one expert considered that the instructions were formulated poorly. The latter expert suggested changes to clarify the instructions, which were later incorporated into the test. A new version of the IFS-Ch was then devised according to these observations. This new version had only minor differences compared with the original test.

*Discriminant validity.* The performance of the two groups differed significantly (p<0.05). Average total scores on the IFS-Ch and each of its subtests were significantly lower in the clinical sample (Table 3). A one-way MANOVA revealed a significant multivariate main effect for diagnosis, Wilks' Lambda=0.225, F_(8, 52)_=22.398, p<0.001, partial eta squared=0.775. The power to detect the effect was 1.00.

**Table 3:** Performance of dementia patients and control subjects in the IFS-Ch and its subtests.

Subtest	Descriptive statistics by group			Comparison	
	**Dementia patients (n=31)**	**Control subjects (n=30)**		**t**	**Significance**
Luria motor series	1.3±1.1	2.8±0.5		7.33	**
Conflicting instructions	1.7±1	2.9±0.3		6.24	**
Go- No go	1.2±0.8	2.3±0.8		5.51	**
Backwards digit span	1.8±1.3	2.9±1		3.92	**
Months backwards	0.6±0.8	1.7±0.7		5.64	**
Modified Corsi tapping test	1.1±0.6	1.7±1		3.16	**
Proverb interpretation	0.7±0.8	2.5±0.5		10.78	**
Modified Hayling test	1.5±1.9	4.8±1.2		8.3	**
Total IFS-Ch	9.8±5.7	21.7±3.4		9.91	**

Results expressed in Mean±Standard Deviation.

** Significant difference, p<0.05. All comparisons were carried out with a t test for independent samples.

The standardized mean differences between the dementia and control groups showed a Cohen's d value (effect size r) of 2.54 (0.79) for the IFS-Ch.

*Convergent and divergent validity.* The total IFS-Ch scores significantly correlated (p<0.05) with other measures of executive functions (categories achieved in the MCST, perseverative errors on the MCST, phonemic verbal fluency with letters A and P, semantic verbal fluency of animals and the FAB); global cognitive efficiency (ACE-R-Ch and MMSE); dysexecutive symptoms (DEX); dementia severity (CDR); and functionality (IADL and T-ADLQ). The coefficients estimated for each association are given in Table 4. The association between IFS-Ch and measures of global cognitive efficiency indicates no evidence of divergent validity.

**Table 4:** Association coefficients between the IFS-Ch and the rest of the administered measures.

	Instrument	IFS-Ch	
		**r_xy_**	**Significance**
Executive functions	FAB	0.935	**
	MCST (categories achieved)	0.791	**
	MCST (perseverative errors)	-0.617	**
	A verbal fluency	0.681	**
	P verbal fluency	0.783	**
	Animals verbal fluency	0.728	**
Global cognitive efficiency	ACE-R-Ch	0.9	**
	MMSE	0.874	**
Dysexecutive symptoms	DEX	-0.494	**
Dementia severity	CDR	-0.75	**
Functional capacity	T-ADLQ	-0.745	**
	IADL	0.717	**

** Significant association, p<0.05. All associations were estimated using a Pearson coefficient, with the exception of the correlation between IFS-Ch and CDR scores, which was executed using a Spearman rank correlation test.

**Evidence of reliability.** The Cronbach's alpha coefficient calculated for the total test was 0.901. Regarding the subtests that included more than one item, the Cronbach's alpha coefficient was 0.577 for the Modified Corsi tapping test, 0.781 for the Proverb interpretation task, and 0.836 for the Modified Hayling test..

**Figure 2:**
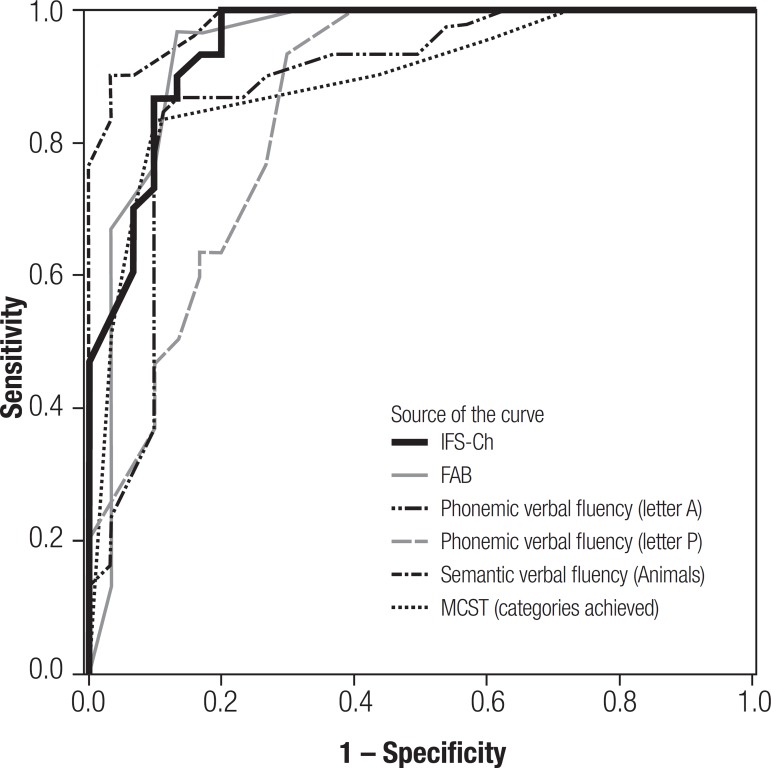
ROC curve for controls vs. patient groups (bvFTD and depression). The superior discriminatory accuracy of the IFS over the MMSE and ACE-R is revealed by its larger area under the curve.

**Diagnostic accuracy.** A ROC curve analysis on the IFS-Ch total score between control subjects and dementia patients generated several cut-off points, with 18 points being the best balance between sensitivity and specificity (sensitivity=0.903; specificity=0.867). The area under the curve (AUC) was 0.951 (Figure 2). There were no significant differences among the areas under the curve of the IFS-Ch, FAB, categories completed on the MCST, Animals verbal fluency, A verbal fluency, and P verbal fluency (p>0.05).^35^

## DISCUSSION

In this paper, the IFS-Ch has shown good psychometric properties and diagnostic accuracy. First, it has shown validity evidence from multiple sources: content validity through expert consultation, discriminant validity by comparing the means of IFS-Ch scores between groups, and convergent validity through associations between IFS-Ch scores and other executive and related measures. Second, the IFS-Ch demonstrated evidence of reliability, exhibiting a good internal consistency coefficient. This is relevant given that reliability is a common weakness of executive tests.^36,37^ With regard to diagnostic accuracy, the selected cut-off point produced an excellent AUC as well as a very good balance between sensitivity and specificity for detecting dementia.

Although the test and two subtests (Modified Hayling test and Proverb interpretation) showed evidence of very good reliability, the Modified Corsi tapping subtest had a poor internal consistency coefficient. Further studies are needed to determine whether this subtest provides an accurate measure of spatial working memory.

It is noteworthy that the cut-off point found in this study^18^ was much lower than that found in the original publication.^25^ This fact is probably due to the socio-demographic differences between both samples, particularly in relation to years of education. In our study, the mean of this parameter was 11.93 and 9.65 years for the control and clinical groups, respectively, whereas in the Argentinian investigation the mean for bvFTD, AD and control subjects was 16.3 years, 14.5 years and 14.5 years, respectively. These differences are coherent with our finding that years of education exhibited a significant association with IFS-Ch total scores and with results of studies showing that education is an important variable in executive test performance in general.^38-40^ Overall, this data suggests that it is important to formulate local norms in order to interpret IFS-Ch scores accurately.

One of the main findings of this study was that the IFS-Ch showed a good association with functionality measures such as the IADL and T-ADLQ. This is coherent with the findings of previous studies which suggest that executive tests predict functional impairment more accurately than tests that assess other cognitive domains,^41-43^ which is expected given that daily life activities are mainly goal-oriented behaviors. Thus, the described association contributes with evidence of convergent validity for the IFS-Ch.

Similarly, a good association was found between IFS-Ch total scores and the DEX, a questionnaire that assesses dysexecutive symptoms. In other words, the IFS-Ch, despite the fact that it is a non-ecological measure - i. e. it is a standardized test administered in a laboratory type setting - correlates significantly with the presence of dysexecutive behaviors in everyday life. The latter constitutes not only evidence of convergent validity for the test, but also suggests that it presents good ecological validity, a relevant and highly desirable feature for an executive assessment instrument.^4,44^

One limitation of our study is the small number of subjects by category of dementia, a situation precluding proper assessment of the capacity of the IFS-Ch to discriminate between different types within the pathology. Moreover, the greater number of patients with AD compared with patients with bvFTD or VD, meant that most of our clinical group presented a multideficit clinical profile, a situation that could explain the significant correlation found between IFS-Ch total scores and measures of global cognitive efficiency (ACE-R-Ch and MMSE). Evidence of divergent validity for the IFS-Ch should be studied with patients presenting deficits mainly in the executive domain. Further research is needed to determine whether the IFS-Ch can differentiate between different forms of dementia and to obtain further evidence of divergent validity.
